# Alphabetical Index to Braithwaite’s Retrospect

**Published:** 1865-03

**Authors:** 


Alphabetical Index to Braithwaite's Retrospect. Embracing Parts 1 to 50—1840—1865. Comprising Twenty-five Years of Republication.

This is a volume of 248 pages, consisting of a full and conveniently arranged Index, of almost inestimable value to all who wish to refer to the past volumes of Braithwaite for the last twenty-five years. Its publication confers a great favor on the Profession. For sale at the Book Stores in this City. Price $1,00.

